# Incidence and Outcomes of Acute Kidney Injury in Critically Ill Patients: A Retrospective Study at a Tertiary Care Hospital

**DOI:** 10.12688/wellcomeopenres.25698.1

**Published:** 2026-03-11

**Authors:** kalpana Kharbuja, Sangina Ranjit, Diptesh Aryal, Subekshya Luitel, Ajit Pandey, Pratikshya Timilsina, Alisha Bajracharya, Sujata Pasachhen, Surendra Bhusal

**Affiliations:** 1 Department of Anaesthesia & critical care, Dhulikhel Hospital, Dhulikhel, Central Development Region, 45210, Nepal; 2 Nepal Intensive Care Research Foundation, Kathmandu, Central Development Region, Nepal; 3 Faculty of Tropical Medicine, Mahidol University,, Mahidol Oxford Tropical Medicine Research Unit, Bangkok, Bangkok, Thailand; 4 D'Or Institute for Research and Education, Rio de Janeiro, State of Rio de Janeiro, Brazil; 5 Department of Anaesthesia and Critical Care, Bir Hospital, Kathmandu, Central Development Region, Nepal

**Keywords:** Acute Kidney Injury (AKI), critically ill patient, morbidity, outcomes

## Abstract

**Background:**

Acute kidney injury is common among critically ill patients and is associated with longer ICU stay, higher resource use and increased mortality. Evidence from low- and middle-income countries, including Nepal, remains limited. This study describes the incidence, severity distribution and clinical outcomes of AKI in a tertiary care ICU.

**Methods:**

We conducted a hospital based retrospective observational study at Dhulikhel Hospital, Nepal. All adults admitted to the ICU from 1 ^st^ may 2023 to 30 ^th^ April 2024 were screened using ICU registry. AKI was defined and staged using KDIGO serum criteria. Demographic data, comorbidities, ICU interventions and outcomes were extracted. Associations were examined using appropriate statistical tests.

**Results:**

Among 1113 patients, 1064 were enrolled for analysis. The incidence of AKI was 29.6%. Patients with AKI were older (median age 60 vs 55 years, p < 0.001), had longer ICU stays (median 3 vs 2 days, p < 0.001), were more frequently on invasive mechanical ventilation (39.6%vs 26%, p < 0.001). Type 2 diabetes mellitus was significantly associated with AKI (39.0% vs 28.0%, p = 0.001). AKI severity correlated with worse outcomes including higher mortality and adverse outcomes increasing from 12.7% in patients without AKI to 38% in stage 3 AKI. Renal replacement therapy was required in 2.3% of patients, mainly in stage 2 and 3 AKI.

**Conclusion:**

Acute kidney injury is a common and a serious complication among critically ill patients, associated with increased mortality and prolonged ICU stay. Early recognition and targeted management strategies, particularly for high-risk groups, are needed in resource limited settings.

## Introduction

Acute kidney injury (AKI), defined by the Kidney Disease: Improving Global Outcome (KDIGO) criteria as abrupt decline in kidney function determined on the basis of increased serum creatinine level and decreased urine output. ^1^ AKI is a common complication of critical illness and independent risk factor for morbidity and mortality. ^2– 4^ AKI rarely occurs in isolation and often results from multiple insults including associated comorbidities as hypertension, diabetes mellitus, hypovolemia, sepsis or septic shock, vasopressor administration, post operative complications after major surgery, trauma, poisioning. ^5– 8^

Developing countries contribute to over 85% of the worldwide incidence of AKI. ^9, 10^ Depending on the AKI definition used, estimates indicate that upto 15% of hospitalized patients and uto 60% those admitted in ICU develop AKI. ^11– 13^ When AKI developed as a episode of sepsis morality report reaches as high as 60%. ^14, 15^ On an average, over 5% of patients with AKI in ICU need RRT. ^12^ It seems that the outcomes perceived in AKI patients in developed countries are different when compared to those from developing countries. ^16^ However, there are very limited studies and data from low- and middle income countries (LMICs). ^17, 18^ To our knowledge, there is little data on the incidence and outcome of AKI in ICU admitted patients in the hospitals of Nepal. A better understanding of the epidemiological profile of AKI in our ICU could open the field for a series of investigations, in order to better understand its relationship with length of hospital stay and mortality, in addition to enabling cost reduction in the healthcare system.

## Methods

### Study design and settings

We conducted a retrospective observational study at the Intensive Care Unit of Dhulikhel Hospital, Nepal. The study period was 1 ^st^ May 2023 to 30 ^th^ April 2024.

### Ethical considerations and consent statement

Ethical approval was obtained from the Institutional Review committee of Kathmandu University of Medical Sciences (IRC-KUSMS Approval No. 278/24, approved August 2024). This study was conducted in accordance with applicable ethical guidelines. This retrospective observational study used anonymised and de-identified ICU patients records collected between 1 ^st^ May 2023 and 30 ^th^ April 2024. The requirement for informed Consent was waived by the ethical committee due to retrospective nature of the study, use of anonymized data, minimal risk and absence of prospective interventions.

Our study received a retrospective ethical approval from the Institutional Review Committee (IRC) at Dhulikhel Hospital (References: (IRC-KUSMS Approval No. 278/24)), dated August 2024. This was granted specifically for retrospective analysis of de-identified routine clinical data, which did not involve prospective recruitment, interventions or contact with patients. Such audits often proceed as quality improvement initiatives under hospital protocols before formal submission as research, however we sought full retrospective approval to manuscript submission to align with publication standards. The IRC confirmed that the minimal risk nature of the study (anonymized secondary data from electronic records with no changes to patient care) justified this approach post data collection.

The IRC waived the need of consent as the study was a retrospective review of de-identified data collected during routine ICU care (study period: 1st May 2023 to 30th April 2024). No identifiable information was assessed, there were no prospective interventions done. This waiver is standard for such low risk observational studies per our institutional guidelines and international norms The IRC waive requirement for informed consent based in accordance with ethical guidelines 2022 of National Health Research Council.

### Study population

All ICU admissions during the study period were screened. Adults aged 18 years or older with an ICU admission during the study window and with available serum creatinine measurements required for AKI assessment were eligible for inclusion. Patients were excluded if they had pre-existing chronic kidney disease, a serum creatinine level greater than 2 mg/dl within the seven days preceding admission, were receiving renal replacement therapy before ICU admission, had AKI diagnosed prior to hospitalization, or had missing essential clinical data.

### Data collection

Data was collected retrospectively from the ICU registry for all patients admitted. Variables were recorded which included patients demographic, comorbidities, clinical parameters, ICU interventions, length of ICU stay and patients outcome. Patient’s electronic and paper medical records were reviewed to extract missing data and confirm AKI diagnosis based on KDIGO criteria.

### Statistical analysis

Data were analyzed using statistical software R version 4.4.3. The incidence of AKI was calculated as the proportion of patients developing AKI during ICU admission. Association between AKI and potential risk factors including comorbidity and clinical interventions, were assessed using univariate analysis. Continuous variables had non normal distribution and expressed as median and interquartile range (IQR). Continuous variables were compared between the two groups using Mann- Whitney U test. Categorical variables were presented as frequency and percentage and compared using Chi-square test or Fischer’s test. AKI staging and its association with outcomes were analyzed to assess the trend of worsening prognosis with increasing AKI severity. Stastical significance was set at p-value <0.05.

## Results

Incidence of AKI = 29.60%.

As shown in Figure 1, a total of 1113 patients were initially screened for study. Six patients were excluded due to missing data. Among them, 23 patients with prior diagnosis of chronic kidney disease (CKD) and 3 patients with serum creatinine >2 mg/dl in previous 7 days were excluded, resulting 1081 patients. Seventeen patients aged less than 18 years were excluded. A total of 1064 patients were eligible and included in analysis.

**Figure 1.**
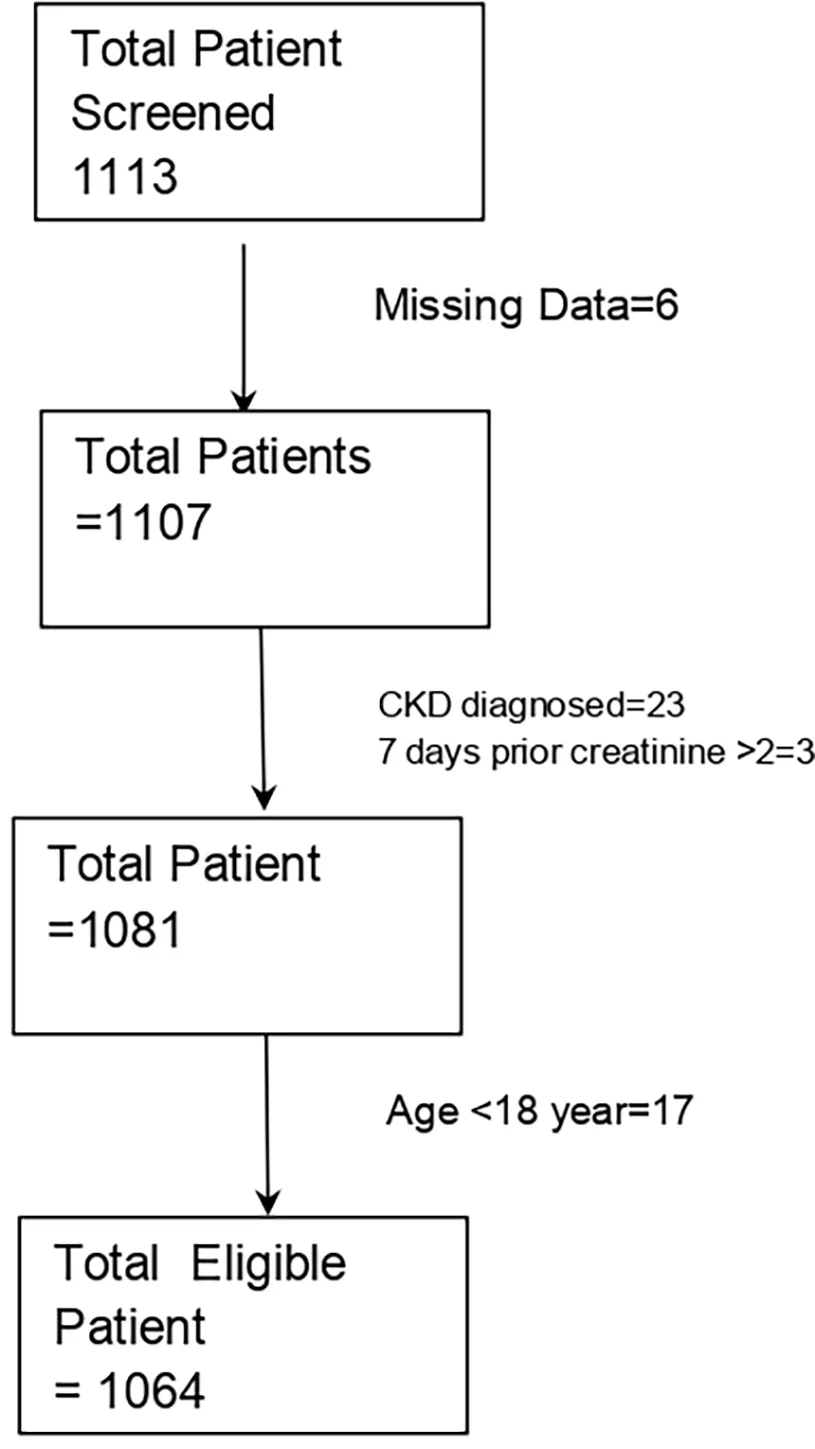
Flow diagram for patient screening.

Table 1 represents the clinical characteristics of patients. Patients with AKI were of older age compared to patients without AKI (Median age 60 vs 55). The median days of ICU stay was longer in patients with AKI compared to patients without AKI (3 days vs 2 days). The duration of mechanical ventilation for patients who developed AKI (Median days = 3) was longer compared to patients without AKI (Median days = 2) but it was not statistically significant. We found that 32.1% of male developed AKI while 26.4% of females developed AKI but the difference was not statistically significant. Among patients with comorbidity, 33.9% developed AKI while among patients without comorbidity, 25.1% developed AKI and the difference was statistically significant. The invasive mechanical ventilation was associated with the incidence of AKI i.e. 39.6% on invasive mechanical ventilation had AKI compared to 26% who did not have invasive mechanical ventilation ( Table 1).

**Table 1.** Clinical characteristics of patients.

Variables		Total patient (N = 1064)	AKI Not diagnosed (n = 749)	AKI diagnosed (n = 315)	p value
AGE (Median (IQR)) years		57 (40–70)	55 (39–69)	60 (44–73)	<0.001
TOTAL ICU STAY (Median (IQR)) Days		2 (1–4)	2 (1–4)	3 (1–5)	<0.001
Total days of Mechanical ventilation (Median (IQR))		2 (1–5)	2 (1–4.5)	3 (1–6)	0.121
Gender	Male	591	401	190 (32.1%)	0.05
	Female	473	348	125 (26.4%)	
Comorbidities	Yes	539	356	183 (33.9%)	0.002
	No	525	393	132 (25.1%)	
Invasive Mechanical ventilation	Yes	283	171	112 (39.6%)	<0.001
	No	781	578	203 (26.0%)	
ICU outcome	Positive	611	471	140 (23.0%	<0.001
	Indeterminate	263	183	80 (30.4%)	
	Adverse	190	95	95 (50%)	

Table 2 illustrates the association between comorbidity and AKI. Out of 1064 patients, hypertension, Type 2 diabetes mellitus, liver disease, COPD, cardiac condition uncontrolled AF and heart block and coronary artery disease were common comorbidity identified. The prevalence of AKI was higher among patients with hypertension (34.2%) vs 28% in non hypertensive (p = 0.06) and those with Type 2 DM (39% vs 28%, p = 0.07). 42.5% of patients with liver disease had AKI and 35.9% of patient with COPD developed AKI where as 37.8% of patients with cardiac conditions developed AKI but only the association with Type 2 DM was statistically significant ( Table 2).

**Table 2.** Association between Comorbidity and AKI.

Comorbidities		Total patient (N = 1064)	AKI Not diagnosed (n = 749)	AKI diagnosed (n = 315)	p value
Hypertension (Count)	Yes	278	183	95 (34.2%)	0.06	
	No	786	566	220(28.0%)
Type 2 DM	Yes	154	94	60 (39.0%)	0.007	
	No	910	655	255 (28.0%)	
Liver disease	Yes	47	27	20 (42.5%)	0.06	
	No	1017	722	295 (29.0%)
COPD	Yes	184	118	66(35.9%)	0.05	
	No	880	631	249 (28.3%)	
Arrhythmia (AF, heart block)	Yes	37	23	14(37.8%)	0.35	
	No	1027	726	301(29.3%)
CAD	Yes	14	10	4(28.6%)	0.99	
	No	1050	739	311(29.61%)	

ICU outcome was classified into three categories. Expiry of patient or leave against medical advice (LAMA) was classified as an ‘adverse outcome’. If a patient was shifted to another ICU (Neurology, medicine, maternal) or other hospital, it was classified as an ‘indeterminate outcome’. If a patient was stepped down such as transfer to HDU, post operative wards or other wards, it was classified as a ‘favorable outcome’. We observed that patients with AKI had more incidence of indeterminate and adverse outcome compared to patients without AKI.

Table 3 summarize the organ system involved and incidence of AKI. The patients with renal disease had the highest incidence of renal failure. Incidence of AKI in patients with sepsis was 54.10%. Third most highest incidence of AKI was seen in cardiovascular disease (39.66%). The least incidence was seen in poisoning, likely because most of the poisoning were OP poisoning ( Table 3).

**Table 3.** Organ system involved and incidence of AKI.

Primary system involved at diagnosis	AKI No (n)	AKI yes (n)	Percentage of patient with AKI (%)	Total patients
Cardiovascular disease	35	23	39.66	58
Endocrine disease	10	5	33.33	15
Gastrointestinal disease	133	68	33.83	201
Musculoskeletal disease	90	24	21.05	114
Neurological disease	173	47	21.36	220
Others	81	30	27.03	111
Poisoning	65	14	17.72	79
Renal disease	7	17	70.83	24
Respiratory disease	127	54	29.83	181
Sepsis	28	33	54.10	61
Total	749	315	29.61	1064

Table 4 presents the ICU outcome by AKI staging. Among 1064 eligible patients, 749 had no AKI (stage 0), 79 had stage 1, 123 had stage2 and 113 had stage 3. For stage 0 (no AKI), 63% had a positive outcome with 12.7% experiencing an adverse outcome. For stage 1, 62% had a positive outcome with 11.4% adverse outcome. For stage 2, only 35% had a positive outcome while adverse outcome was observed in 35%. For stage 3, positive outcome was observed in 42% and adverse outcome increased to 38%. As AKI severity progressed, there was a corresponding increased in fraction of patients with adverse outcomes ( Table 4).

**Table 4.** ICU outcome by AKI staging.

AKI staging	Total patients	Positive outcome n(%)	Indeterminate outcome n(%)	Adverse outcome n(%)
0	749	471 (63%)	183 (24.3%)	95 (12.7%)
1	79	49 (62%)	21 (26.6%)	9 (11.4%)
2	123	43 (35%)	37 (30%)	43 (35.0%)
3	113	48 (42%)	22 (20%)	43 (38.0%)

As shown in the Figure 2, patients without AKI with stage 1 AKI did not required RRT. In stage 2, 6 patients (4.9%) required RRT while majority did not required. In stage 3, 19 patients (16.8%) received RRT and total 25 patients (2.3%) required RRT ( Figure 2).

**Figure 2.**
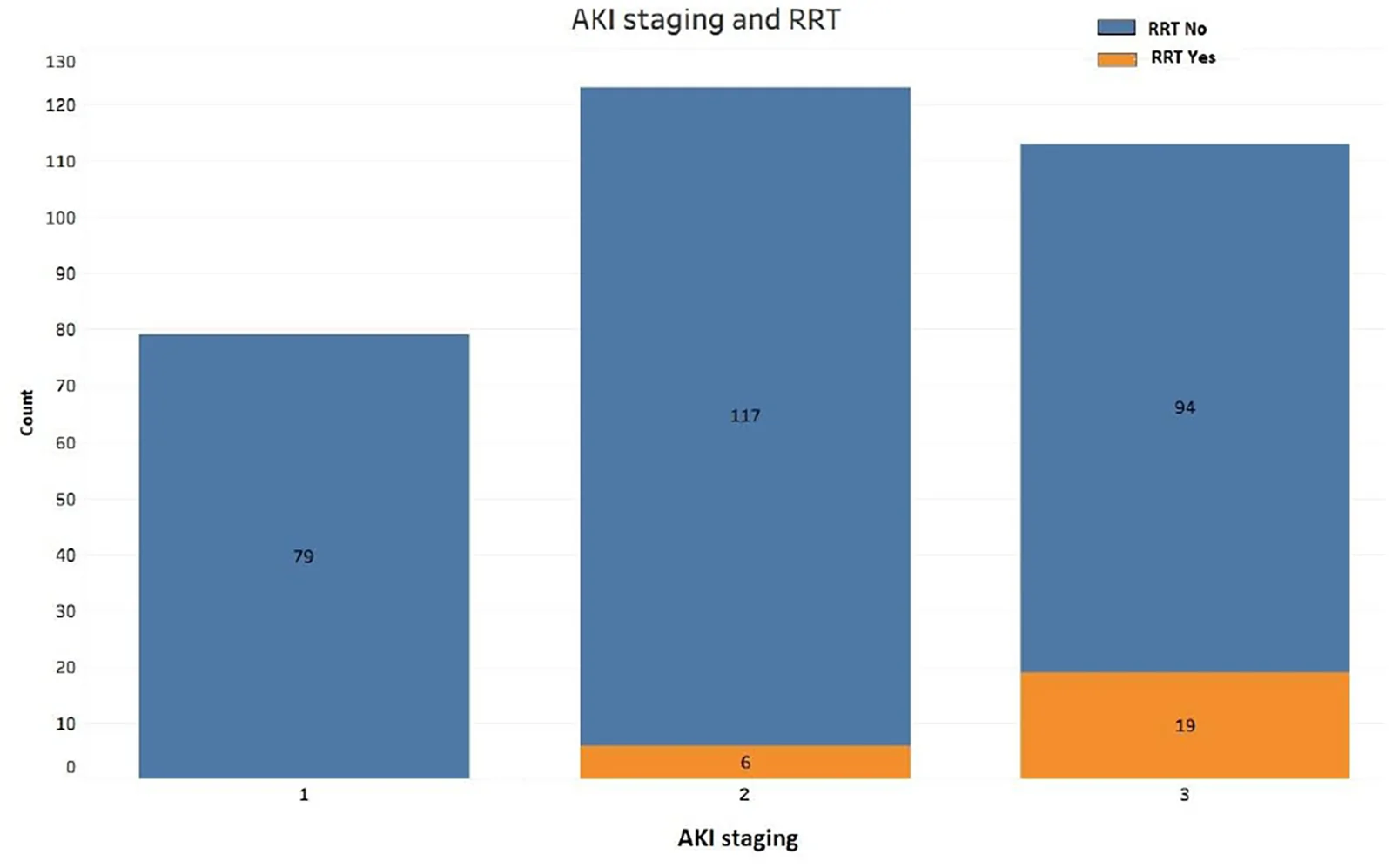
AKI staging and RRT.

Table 5 represents the ICU outcome. Among 1064 patients, 749 without AKI (70.4%) and 315(29.6%) with AKI were analysed for ICU outcome. Discharge rate was higher in the non AKI group 24 vs 13 (64.9% vs 35.1%). Mortality was higher in patients with AKI, with 50 deaths (15.9%) compared to 37 (4.9%) in the non-AKI group. Fifty eight patients (7.74%) without AKI and 45 (14.3%) with AKI left against medical advice. Referrals to other hospitals and transfers to high dependency units were relatively low in both groups. A large proportion of patients without AKI were shifted to post-operative wards (188 vs 46) and general wards (244 vs 73) compared to those with AKI. These findings indicate that AKI is associated with increased mortality and less favourable discharge outcomes ( Table 5).

**Table 5.** ICU outcome.

ICU outcome	AKI		Total
	No (n)	Yes (n)	
DISCHARGE	24 (64.9%)	13(35.1%)	37
EXPIRED	37 (4.9%)	50 (15.9%)	87
LAMA	58(7.74%)	45(14.3%)	103
REFER TO OTHER HOSPITAL	2 (0.3%)	3(0.9%)	5
SHIFTED TO HDU	15(2%)	8(2.5%)	23
SHIFTED TO OTHER ICU	181(24.2%)	77(24.4%)	258
SHIFTED TO POST OP WARD	188 (25.1)	46 (14.6)	234
SHIFTED TO WARDS	244 (32.6%)	73 (23.2%)	317
Total	749 (70.4%)	315 (29.6%)	1064

## Discussion

In this single centre cohort of critically ill adults, the incidence of acute kidney injury was 29.6 percent. This rate is similar to reports from other low- and middle-income countries. Prior work from Nepal described a lower incidence of 14.2 percent, ^19^ while studies from settings such as Jordan and Sudan reported higher rates between 31 and 35 percent. ^20, 21^ Such variation is common and is influenced by differences in patient characteristics, severity of illness, accuracy of baseline creatinine data, and thresholds for intensive care admission. When considered alongside large international datasets, where acute kidney injury incidence ranges from 21 to 57 percent in high income settings and reached 53.5 percent in the multinational AKI EPI study, the burden observed in our cohort appears consistent with global patterns seen in resource limited environments. ^22– 26^

Several clinical factors were associated with the development of acute kidney injury in our patients. Older age and the presence of comorbidities such as diabetes, hypertension, liver disease, and chronic lung disease were more common among those who developed renal injury. These findings are aligned with previous research showing that metabolic disease, chronic inflammatory states, and vascular dysfunction increase susceptibility to renal injury during critical illness. ^22, 23, 27, 28^ The significant association between diabetes and acute kidney injury in our cohort is noteworthy. Diabetes is known to impair renal autoregulation and microvascular function, which reduces the kidney’s ability to tolerate hemodynamic stress. The high incidence of acute kidney injury among patients with underlying renal disease, sepsis, and cardiovascular conditions further reflects well established biological pathways linking systemic inflammation, circulatory failure, and nephrotoxic exposures to acute renal injury. ^29^

The clinical trajectory of patients with acute kidney injury in our study also followed patterns described in the international literature. ^22, 28– 31^ Patients with acute kidney injury stayed longer in the intensive care unit, required invasive mechanical ventilation more often, and experienced progressively worse outcomes as severity increased. This dose response pattern is well recognized. As acute kidney injury progresses through KDIGO stages, renal perfusion deficits, tubular injury, and systemic inflammation intensify, leading to increased organ dysfunction. Although the overall proportion of patients requiring renal replacement therapy was lower than that reported in many high-income settings, the increase in dialysis requirement from stage 2 to stage 3 was expected. The lower overall use of renal replacement therapy in our setting probably reflects more restrictive initiation practices, limited availability of dialysis modalities, and careful selection of candidates for renal support.

The high mortality among patients with acute kidney injury highlights the vulnerability of critically ill populations in resource constrained environments. Several local factors may contribute to poorer outcomes, including delayed presentation to hospital, late admission to intensive care, limited monitoring capacity, and restricted access to renal replacement therapy. The absence of early detection tools such as biomarkers or automated alert systems may further delay recognition and timely management. The combined effect of a high burden of comorbidity and constrained critical care resources likely magnifies the impact of acute kidney injury on survival in Nepal.

These findings have important implications for clinical practice. Focusing surveillance on high-risk groups, particularly older adults, patients with diabetes, and those requiring mechanical ventilation, may support earlier recognition. Strengthening essential practices such as careful fluid management, avoidance of nephrotoxic medications, and the use of standardized acute kidney injury care bundles may offer a practical and cost-effective pathway to improvement. Expanding access to renal replacement therapy and promoting timely referral may also play a significant role in reducing preventable mortality.

This study underscores the need for prospective, multi-centre research to better define the epidemiology of acute kidney injury in Nepal. Long term follow up is essential to understand renal recovery, new onset chronic kidney disease, and the risk of recurrent renal injury. Future studies should also evaluate the impact of simple prevention and treatment strategies that are feasible in low resource intensive care environments.

## Conclusion

Acute kidney injury was common in our intensive care population and was strongly associated with longer ICU stay, higher need for organ support, and increased mortality. Older patients and those with diabetes were particularly vulnerable. These findings highlight the need for earlier recognition of acute kidney injury and improved supportive care practices in resource constrained settings.

### Strength

This study benefits from a large sample size and the use of a structured registry, which allowed comprehensive and consistent data collection.

### Limitation

The single centre design limits generalisability, and the retrospective nature of the study introduces potential bias due to missing or incomplete data. The lack of long term follow up also prevents assessment of renal recovery or progression to chronic kidney disease.

## Data Availability

Figshare: Incidence and Outcomes of Acute Kidney Injury in Critically Ill Patients: A Retrospective Study at a Tertiary Care Hospital https://doi.org/10.6084/m9.figshare.30962276.V1 ^32^ The project contains the following underlying data: Incidence and Outcomes of Acute Kidney Injury in Critically Ill Patients_A Retrospective Study at a Tertiary Care Hospital. Figshare: Incidence and Outcomes of Acute Kidney Injury in Critically Ill Patients: A Retrospective Study at a Tertiary Care Hospital https://doi.org/10.6084/m9.figshare.30962276.V1 ^32^ The project contains the following extended data: Incidence and Outcomes of Acute Kidney Injury in Critically Ill Patients_A Retrospective Study at a Tertiary Care Hospital_CRF_2026. Data are available under the terms of the Creative Commons Attribution 4.0 International license (CC-BY 4.0).
