# Supplementary material for: Incidence and Outcomes of Acute Kidney Injury in Critically Ill Patients: A Retrospective Study at a Tertiary Care Hospital
Source: Wellcome Open Res. 2026 Oct 13;11:161. Originally published 2026 Mar 11. [Version 2] doi: 10.12688/wellcomeopenres.25698.2 (PMC13714418; doi:10.12688/wellcomeopenres.25698.2)
Supplement: Peer review history

# Reviewer report: Reviewer response for version 2

**DOI:** 10.21956/wellcomeopenres.30761.r173820

**Published:** 2026-10-27

**Authors:** Jie Tang

**Affiliations:** 1 Division of Kidney Diseases and Hypertension, The Warren Alpert Medical School of Brown University, Providence, Rhode Island, USA

Recommend: indexing

Is the work clearly and accurately presented and does it cite the current literature?

Yes

If applicable, is the statistical analysis and its interpretation appropriate?

Yes

Are all the source data underlying the results available to ensure full reproducibility?

Yes

Is the study design appropriate and is the work technically sound?

Yes

Are the conclusions drawn adequately supported by the results?

Partly

Are sufficient details of methods and analysis provided to allow replication by others?

Partly

Reviewer Expertise:

Genetics, clinical epi, global health

I confirm that I have read this submission and believe that I have an appropriate level of expertise to confirm that it is of an acceptable scientific standard.

# Reviewer report: Reviewer response for version 1

**DOI:** 10.21956/wellcomeopenres.28296.r166624

**Published:** 2026-09-09

**Authors:** Batsalya Arjyal

**Affiliations:** 1 Birat Medical College and Teaching Hospital, Biratnagar, Nepal

This retrospective single-center observational study evaluated the incidence, clinical risk factors, and short-term outcomes of acute kidney injury (AKI) among 1,064 adult critically ill patients admitted to Dhulikhel Hospital, Nepal, over a 12-month period from May 2023 to April 2024. The study used the serum creatinine component of the KDIGO criteria and found that AKI occurred in 29.6% of critically ill patients.

 The study identified several factors associated with the development of AKI. Older age, the presence of pre-existing comorbidities—particularly type 2 diabetes mellitus—and the need for invasive mechanical ventilation were significantly associated with AKI on crude analysis. Increasing severity of AKI, from KDIGO stage 1 through stage 3, was also associated with longer ICU stays and poorer clinical outcomes, including mortality and discharge against medical advice. Only 2.3% of the study population received renal replacement therapy.

 The study is particularly relevant because it contributes to the limited epidemiological evidence on AKI in critically ill patients from low- and middle-income countries.

 The relatively large sample size add to the data that will help clinicians and healthcare planners better understand the burden of AKI and its implications for ICU resource utilization in critical care setup of Nepal.

Is the work clearly and accurately presented and does it cite the current literature?

Yes

If applicable, is the statistical analysis and its interpretation appropriate?

I cannot comment. A qualified statistician is required.

Are all the source data underlying the results available to ensure full reproducibility?

Yes

Is the study design appropriate and is the work technically sound?

Yes

Are the conclusions drawn adequately supported by the results?

Yes

Are sufficient details of methods and analysis provided to allow replication by others?

Yes

Reviewer Expertise:

critical care

I confirm that I have read this submission and believe that I have an appropriate level of expertise to confirm that it is of an acceptable scientific standard.

# Reviewer report: Reviewer response for version 1

**DOI:** 10.21956/wellcomeopenres.28296.r166623

**Published:** 2026-09-02

**Authors:** Jie Tang

**Affiliations:** 1 Division of Kidney Diseases and Hypertension, The Warren Alpert Medical School of Brown University, Providence, Rhode Island, USA

This is a retrospective, single-center study reporting the incidence, clinical characteristics, and outcomes of AKI in the ICU setting. The study addresses an important gap in the literature and will provide much-needed clinical data on AKI in LMICs, including Nepal.

**Study design/Methods**

 - The authors exclude patients with CKD. I wonder why this exclusion criterion was used, as AKI superimposed on CKD is common and represents an important patient population in which there is a significant need to optimize care.

 - Among the 1,113 screened study participants, only 23 were reported to have baseline CKD. This proportion seems unusually low and warrants clarification. How many patients in the study population had pre-existing clinical or laboratory data available? In many LMICs, patients do not routinely receive outpatient care, and the lack of electronic medical record systems makes it even more difficult to retrieve historical data. Please clarify whether complete historical clinical and laboratory data were available and reviewed for all 1,064 participants included in the final analysis. In addition, what criteria were used in this study to define and exclude baseline CKD (needs to be stated in the method section)? 

 - Please consider including key potential confounding factors related to the severity of the underlying acute illness that led to ICU admission, such as sepsis, shock, acute myocardial infarction, heart failure, ARDS, or decompensated cirrhosis. Adjustment for these factors would help clarify whether AKI is independently associated with the reported outcomes or primarily reflects the severity of the underlying disease process. Authors did mention some of the admission diagnoses, but please provide details. 

 Please correct the typographical errors and consider using an English-language editing service to further improve the manuscript.

 Example: In the introduction section, "Depending on the AKI definition used, estimates indicate that upto 15% of hospitalized patients and uto 60% those admitted in ICU develop AKI." - uto should be up to...

**Ethical considerations and consent statement:**

 Please consider shortening this section, as the messages are repetitive and there is too much unnecessary verbiage.

**Discussion:**

 Based on the methods described, all patients developed AKI during their ICU stay. Please be cautious about implying or drawing conclusions regarding the independent effect of AKI on the reported outcomes. AKI may instead be a marker of underlying disease severity that contributed to the poor outcomes, rather than a causal factor.

Is the work clearly and accurately presented and does it cite the current literature?

Yes

If applicable, is the statistical analysis and its interpretation appropriate?

Yes

Are all the source data underlying the results available to ensure full reproducibility?

Yes

Is the study design appropriate and is the work technically sound?

Yes

Are the conclusions drawn adequately supported by the results?

Partly

Are sufficient details of methods and analysis provided to allow replication by others?

Partly

Reviewer Expertise:

Genetics, clinical epi, global health

I confirm that I have read this submission and believe that I have an appropriate level of expertise to confirm that it is of an acceptable scientific standard, however I have significant reservations, as outlined above.
